# Bone-softening

**Published:** 1896-04

**Authors:** M. D. Hoge

**Affiliations:** Richmond, Va.


### BONE–SOFTENING.[1]

By M. D. HOGE, Jr., M.D.,
RICHMOND, VA.

As the diseases osteoporosis and osteomalacia in animals are among the most obscure, and our Society having entered upon their investigation, I have thought that a short review of kindred diseases as observed in man might from analogy give us some possible insight into the cause of these mysterious troubles.

[1] A paper read before the Virginia State Veterinary Medical Association, January 2, 1896.

Rhachitis, or rickets, was first accurately described by an Englishman, Glisson, in 1650. It first made its appearance in England about the beginning of the seventeenth century, and even to this day it is called by the Germans *die Englische Krankheit*, "the English disease."

The true and real cause is at present unknown, in spite of the great number of careful observations and painstaking investigations. It is, however, certain that its development is promoted by defective nourishment, unsanitary surroundings, as damp, crowded quarters in large cities, and in artificially fed children.

It has been found possible to produce the disease, or certain similar changes, in the bones of young animals by giving foods as free from lime as possible, and by the administration of large doses of lactic acid or small quantities of phosphorus. The changes thus produced bear an analogy to rhachitis, or rather to bone-softening. Still the specific etiological factor is wanting.

Some authorities contend that the disease bears some relation to syphilis; others that it is either a peculiar form of malacia, or the results of the same. It generally appears in children from two to three years of age, among the wealthy as well as the poor, and sex has no influence on its occurrence.

Rhachitis is a disturbance of the normal process connected with the growth of bone. It is not that they *become* soft, but they remain soft, *i.e.*, not ossified. We know that all the bony structures of the fœtus, except the vault of the cranium, are mapped out in hyaline cartilage. Pathologically, we find the periosteum much reddened and congested. In tearing off the periosteum small pieces of the unossified tissue adhere to it. In normal (long) bones, at the epiphyses, the proliferative layer or hyperplastic zone is sharply and distinctly separated from the osseous layer or zone of calcification. In rhachitic bone the sharp boundaries are replaced by an irregular serrated edge. The prolification of the cartilage cells has increased beyond all bounds, and the cartilage matrix has become fibrous. There is also an extremely active new growth of bloodvessels. The periosteum shows changes also; the inner or osteoblastic layer is thickened, and the newly formed bone does not become completely calcified, but remains soft and spongy. Hence we have swelling of the epiphyses of the long bones and thickening of the flat bones, especially those of the skull.

Normal dry bone contains about 63 per cent. of lime; rhachitic bones have only 20 per cent. to 30 per cent. Experimental proof on animals shows us that when the lime-salts are withdrawn from the food of ewes the lambs suffer from rhachitis. In zoölogical gardens, young lions and leopards also have rhachitis when the bones are first cut off from the fresh meats fed to them.

To state the views again as to the causes of rhachitis, most authors maintain that it is a disturbance of nutrition in which the lime-salts are deficient. Kassowitz seeks to explain the cause by a pathologically increased vascularization of the osteogenetic tissue, in which the bloodvessels are especially vulnerable by reason of deficient nutrition and toxic substances in the circulation.

In other cases the lime-salts are in abundance, but, owing to a catarrhal condition of the intestines, they are not absorbed, hence do not reach the osseous tissue. According to Salkowski and Seemann, lime-salts in *excess* have a similar effect, in that phosphate of lime combines with the chlorine of the blood, thereby producing a deficiency of chlorides. The result of this is a deficiency of hydrochloric acid in the stomach, and by this means the solubility and absorption of lime-salts are made impossible.

We may still conceive that there exists, undiscovered as yet, in the blood a toxin which has the power of exciting in the bones the previously described methods of abnormal growth, osteomalacia.

As a rule osteomalacia does not, like rhachitis, consist of a disturbance in development of bone, but the bones, having already undergone normal development and hardness, afterward become soft. Its true etiological factor has as yet not been ascertained. It is especially frequent along the Rhine, in Westphalia, Flanders, and Northern Italy. This might point to some local infection in these localities which is yet undiscovered.

Among the exciting causes child-bearing is the most important, for the disease usually dates from pregnancy. Another factor which may be mentioned is unhygienic surroundings. The pathological process consists in a disappearance of the earthy salts of the bones, which begins usually from the medulla and extends outward. The marrow is at first extremely hyperæmic and extravasations of blood are frequently found. The bony structure surrounding the Haversian canals becomes converted into a soft fibrous tissue, the central cavity grows larger and larger, and the cortical substance becomes as thin as paper.

At this stage the hyperæmia disappears and the marrow acquires a yellow color, which may be entirely transformed into a yellow viscid fluid. The bones can now be easily cut with a knife; they possess a low specific gravity. The periosteum is at first thickened and hyperæmic, and when it is removed the underlying surface of the bone is found rough and uneven.

Chemically there is a large deficiency of lime-salts, and lactic acid is found in the bones. The general condition of the patient, except for intense pain, remains good for a long time; the appetite is good and the internal organs perform their functions in a normal manner.

The excretion of phosphates in the urine is increased; lactic acid has been found. Concretions of lime are met with in the bladder and kidneys.

